# Impact of empathy level on nurses’ attitudes toward a suicidal patient

**DOI:** 10.1192/j.eurpsy.2023.2354

**Published:** 2023-07-19

**Authors:** B. Amamou, R. Manaa, M. Ben Mbarek, T. Jarray, F. Zaafrane, L. Gaha

**Affiliations:** Psyciatry, faculty of medicine of Monastir, university of monastir, Monastir, Tunisia

## Abstract

**Introduction:**

The number of suicidal patients who are consulting hospitals is increasing. Therefore nurses are more frequently faced to dealing with these patients. Empathy plays an essential role in the nurse-patient relationship and influences therapeutic effectiveness.

**Objectives:**

Assess the impact of empathy level and investigate its factors on psychiatric and emergency department nurses’ attitudes toward a suicidal patient.

**Methods:**

This is a cross-sectional analytical study that focused on nurses working in emergency and psychiatric departments in two Tunisian Hospitals. It was conducted between February and April 2022.

We used the Cognitive and Affective Empathy questionnaire (QCAE) to evaluate the empathy level.

**Results:**

Our study involved 60 nurses with an average age of 35,23 and a sex ratio of 0.76. Forty-seven percent of the nurses feel pity for the suicidal person while 16.7% remain indifferent.

Fifty-seven percent of respondents believe that attempting suicide is primarily a sign of weakness and 45% believe that its a sign of suffering.

While dealing with a suicidal patient, 45% of caregivers choose to reassure family and friends while 6.7% prefer to call the police to investigate.

Among the nurses, 58.4% had an affective empathy score greater than or equal to 30 while 51.9% of them had a cognitive empathy score greater than or equal to 40.

There was no statistically significant association (p>0.05) between the QCAE score and: age, gender, marital status, number of years in current service, number of children, and personal psychiatric history.

There is a significant association between The department and both emotional Contagion and Perspective taking ( p<0.05), while no significant association between Proximal Responsiveness, Peripheral Responsiveness, and Online Simulation.

**Conclusions:**

It is undeniable that empathy level affects the quality of the relationship between suicidal patients and caregivers with many influencing factors.

The nurse has a therapeutic role but also a preventive one with regard to the problem of suicide, Therefore, the training of medical and paramedical teams is essential in order to limit any negative attitudes.

**Disclosure of Interest:**

None Declared

